# Generation of iPSC line from a Parkinson patient with *PARK7* mutation and CRISPR-edited Gibco human episomal iPSC line to mimic *PARK7* mutation

**DOI:** 10.1016/j.scr.2021.102506

**Published:** 2021-08-17

**Authors:** Melissa Conti Mazza, Alexandra Beilina, Dorien A. Roosen, David Hauser, Mark R. Cookson

**Affiliations:** National Institute on Aging, National Institutes of Health, Bethesda, MD 20892, United States

## Abstract

Mutations in the oncogene *PARK7*, which codes for DJ-1, have been associated with early-onset autosomal recessive Parkinson’s disease (PD); however, the exact role of DJ-1 in PD remains elusive. Fibroblasts from a PD patient with a uniparental disomy, 1 bp deletion in *PARK7* were reprogrammed into the induced pluripotent stem cell (iPSC) line: NIHTVBi015-A. For control purposes, CRISPR-Cas9 editing was used to mimic the mutation in the Gibco Human Episomal iPSC line: TMOi001-A is the control line (A18945) and TMOi001-A-3 is the control-edited line (2B10). All 3 lines exhibit normal karyotyping and expression of pluripotent markers: OCT4, SOX2, and NANOG. These lines provide a translational environment to study DJ-1-related function in PD.

## 1. Resource table

**Table:** 

Unique stem cell lines identifier	1. NIHTVBi015-A
	2. TMOi001-A-3
	3. TMOi001-A
Alternative names of stem cell lines	4. HT188 (NIHTVBi015-A)
	5. 2B10 (TMOi001-A-3)
	6. A18945 (TMOi001-A)
Institution	1. NIH National Heart, Lung, and Blood Institute IPSC Core
	2. National Institutes of Health
	3. Commercially available
Contact information of distributor	Mark Cookson, cookson@mail.nih.gov
Type of cell lines	iPSC
Origin	Human
Cell Source	1. Fibroblasts
	2. CD34 + cord blood
	3. CD34 + cord blood
Clonality	Clonal
Method of reprogramming	1. Sendai virus
	2. Three-plasmid, seven-factor EBNA-based episomal system
	3. Three-plasmid, seven-factor EBNA-based episomal system
Multiline rationale	Control and disease pair
Gene modification	1. No
	2. Yes
	3. No
Type of modification	Induced mutation
Associated disease	Parkinson’s disease
Gene/locus	NM:001123377.1 (PARK7): Chr1(GRCh37): g.8037720del; p.Ala111Leufs*7
Method of modification	CRISPR
Name of transgene or resistance	N/A
Inducible/constitutive system	N/A
Date archived/stock date	N/A
Cell line repository/bank	N/A
Ethical approval	1. NIH Undiagnosed Diseases Program, 76-HG-0238

## 2. Resource utility

The *PARK7* gene encodes for DJ-1 and causes an autosomal recessive early-onset form of Parkinson’s disease (Bonifati et al., 2003). The iPSC line from a PD patient with a *PARK7* loss-of-function mutation, and the line mimicking the mutation in the A18945 control line, are valuable tools in which to study DJ-1-related mechanisms of neurodegeneration.

## 3. Resource details

**Fig. 1.**
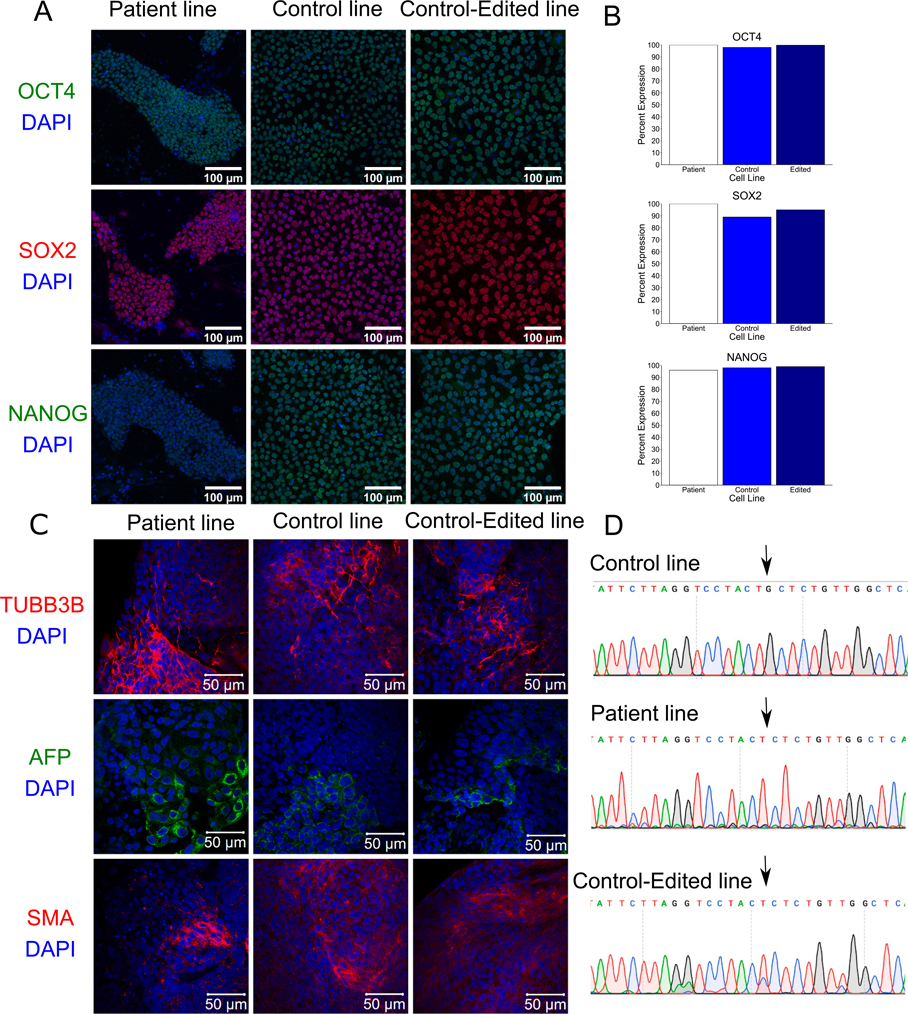


To date, all *PARK7* mutations related to PD are loss-of-function mutations (Hauser et al., 2016). Fibroblasts were taken from an early-onset PD patient carrying a homozygous single base pair deletion in the *PARK7* gene (c.331del (p.A111L); Table 1). Fibroblasts were converted to the pluripotent state (patient iPSC line HT188 (NIHTVBi015-A)) using the Sendai method of reprogramming (NIH National Heart, Lung, and Blood Institute IPSC Core). We tried to correct the mutation using CRISPR-editing techniques, but low transfection rates prevented this. Therefore, we inserted the mutation into the commercially available control iPSC line A18945 (Gibco; (TMOi001-A)) using CRISPR-editing (edited iPSC line 2B10 (TMOi001-A-3)) so to have a genetically matched control line. All lines have normal morphology, but the patient iPSC line needs to be seeded onto mouse embryonic fibroblasts (MEFS) to survive (Supplementary Fig. 1A). Pluripotency was confirmed through the detection of OCT4, SOX2, and NANOG at the protein level using immunocyctochemistry (Fluorescent Human ES/iPS Cell Characterization Kit, Sigma-Aldrich, SCR078; Fig. 1A–B). All three lines were able to undergo embryoid body differentiation and express markers for the three layers including beta-tubulin (TUBB3B; ectoderm), alpha-feto protein (AFP; endoderm), and muscle actin (SMA, mesoderm; Fig. 1C). All three lines have normal karyotyping with no detectable clonal abnormalities at the stated level of band resolution (Table 2, Supplemental Fig. 1B; WiCell). According to short tandem repeat (STR) analysis (WiCell), all lines were unique and not contaminated with other cell lines. However, while the patient iPSC line did not display allelic imbalances, the control and edited iPSC lines did express allelic imbalances which may be due to chromosomal gains, losses, and/or amplifications (see journal archive). The control iPSC line was matched with four other cell lines that were generated from it, including the edited iPSC line (Table 2). All lines were sequenced to verify that the guanine at c.331 locus was either present (control line, arrow) or absent (patient and edited lines) in a homozygous fashion (Fig. 1D). To detect potential off-target effects of the deletion and CRISPR-editing, all lines were submitted for whole genome sequencing (WGS, Psomagen). No off-target effects were identified in the top seven predicted sites (Supplementary Fig. 1D). Sequences for all three lines have been made available (Table 2). Finally, all lines tested negative for mycoplasma (Supplementary Fig. 1C) (see Table 3).

## 4. Materials and methods

### 4.1. Reprogramming

For the patient HT188 line, fibroblasts were reprogrammed following the Sendai Cytotune 1 (A1378001) and Cytotune 2 (A16517) virus kit (Life Technologies) instructions by NIH National Heart, Lung, and Blood Institute IPSC Core as previously described (Beers et al., 2015). The Gibco Human Episomal iPSC Line (A18945; ThermoFisher Scientific) was commercially purchased and derived from CD34+ cord blood using the three-plasmid, seven-factor EBNA-based episomal system.

### 4.2. CRISPR editing

CRISPR editing was used to create the edited 2B10 line by inserting the single base pair mutation into the commercially available control A18945 line to mimic the patient-derived HT188 line. gRNA was cloned into the pSP-Ef1α-Cas9–2A-GFP plasmid backbone. Cells were transfected with the Cas9/gRNA plasmid (pSP-Ef1α-Cas9–2A-GFP; Integrated DNA Technologies) and donor oligo using lipofectamine™ stem transfection reagent (Thermo Fisher Scientific). Cells were prepped for fluorescence-activated cell sorting (FACS) sorting for GFP-positive cells then expanded until recovered. Cells were then treated with Accutase (Thermo Fisher Scientific) for a single-cell suspension and seeded to generate colonies for monoclonal lines. Once colonies had recovered, the Guide-it Mutation Detection kit (Takara) was used to prep samples for PCR amplification and sequencing analysis by Psomagen for verification of the mutation. gRNA, donor oligo and diagnostic primers to verify mutation insertion are listed in Table 2.

### 4.3. Karyotyping

Karyotyping was performed by the WiCell Characterization Lab (Madison, WI). Cells were seeded in a T25 flask and shipped 3 days after passaging (HT188 at p8, A18945 at p4, 2B10 at p5) when at ~50% confluency.

### 4.4. Short tandem repeat (STR) analysis

STR analysis was carried out by the WiCell Characterization Lab (Madison, WI). Cells were seeded in a T25 flask and shipped 3 days after passaging (HT188 at p4, A18945 at p4, 2B10 at p5) when at ~50% confluency.

### 4.5. Whole genome sequencing (WGS)

Cells (passage 4–5) underwent DNA extraction using the 25:24:1 Phenol:Chloroform:Isoamyl alcohol protocol. The resulting DNA pellet was resuspended in autoclaved water then sent to Psomagen for WGS and off-target analysis.

### 4.6. Mutation analysis

To verify the presence and absence of the mutation, all three lines were sequenced using the Sanger sequencing method, respectively. The DNeasy blood and tissue kit (Qiagen) was used to extract gDNA for PCR amplification with Terra PCR polymerase mix (Takara). AMPure purification reagents (Beckman) and sequencing using BigDye on a 3730 DNA Analyzer (Applied Biosystems) followed. Primers to verify mutation insertion are listed in Table 2.

### 4.7. Immunofluorescence staining

Immunofluorescence staining was carried out following the Fluorescent Human ES/iPS Cell Characterization Kit (Millipore Sigma, SCR078) protocol. Antibody information is listed in Table 2. Images taken on a Zeiss 780 confocal microscope.

### 4.8. Embryoid body (EB) formation

iPSC lines were dissociated into single cells with TrypLE™ Express (Life Technologies), and 10,000 cells/well were plated into low-cell-adhesion 96-well culture plates with V-bottomed conical wells (Sumitomo Bakelite) to form uniform EBs cultured in: DMEM media, 20% FBS, 1 mM L-glutamine, 0.1 mM β-mercaptoethanol, 1% nonessential amino acids, P/S. After 20 days, EBs were fixed with 4%PFA and stained for three markers: smooth muscle actin (mesoderm), beta-III Tubulin (ectoderm), alpha-fetoprotein (endoderm) by Molecular Probes® 3-Germ Layer Immunocytochemistry Kit (Cat. No. A25538).

### 4.9. Mycoplasma detection

Mycoplasma testing was performed by PCR (Uphoff and Drexler, 2004). Cell lines (passage 4–5) were scraped in PBS and spun down to remove supernatant. Pellets were lysed using proteinase K (1 mg/mL) and DirectPCR lysis reagent (Viagen Biotech). Detection primers is listed in Table 2.

## Supplementary Material

1

## Figures and Tables

**Table 1:** Summary of lines.

iPSC line names	Abbreviation in figures	Gender	Age	Ethnicity	Genotype of locus	Disease

HT188 (NIHTVBi015-A)	HT188	Female	35	Northern European	Homozygous PARK7 c.331del (p.A111L)	Parkinson’s disease
2B10 (TMOi001-A-3)	2B10	Female	Unknown	Unknown	Homozygous PARK7 c.331del (p.A111L)	Mimics mutation
A18945 (TMOi001-A-3)	A18945	Female	Unknown	Unknown		Commercially available control line

**Table 2:** Characterization and validation.

Classification	Test	Result	Data

Morphology	Photography	Normal	Supplementary Fig. 1A
Phenotype	Qualitative analysis	Verified expression of OCT4, SOX2, and NANOG	Fig. 1A
	Quantitative analysis	Assess % of positive cells for antigen markers: OCT4: all above 98%, SOX2: all above 89%, NANOG: all above 96%	Fig. 1B
Genotype	Karyotype (G-banding) and resolution	1. 46,XX Resolution 500–550 2. 46,XX Resolution 450–575 3. 46,XX Resolution 425–500	Supplementary Fig. 1B
Identity	Microsatellite PCR (mPCR) OR STR analysis	Not performed 16 loci tested: 1. No matches 2. No matches 3. Four matches to other cell lines derived from this commercial line	N/A Submitted in archive with journal
Mutation analysis (IF APPLICABLE)	Sequencing	Homozygous, c.331del	Fig. 1D
	WGS	No off-target effects.	Sequencing deposited at Sequence Read Archive: SRA accession PRJNA622304. Release date: 2020-09-01. https://www.ncbi.nlm.nih.gov/sra/PRJNA622304 Supplementary Fig. 1D
Microbiology and virology	Mycoplasma	Mycoplasma testing by RT-PCR. All negative	Supplementary Fig. 1C
Differentiation potential	Embryoid body formation	Expression of muscle actin, β-III tubulin and α-feto protein.	Fig. 1C
Donor screening (OPTIONAL)	HIV 1 + 2 Hepatitis B, Hepatitis C	Not performed	N/A
Genotype additional info (OPTIONAL)	Blood group genotyping	Not performed	N/A
	HLA tissue typing	Not performed	N/A

**Table 3:** Reagents details.

Antibodies used for immunocytochemistry/flow-citometry			
	Antibody	Dilution	Company Cat # and RRID

Pluripotency Marker	Mouse anti-Oct-4, Alexa Fluor® 488 conjugate	1:100	Millipore Sigma, MAB4419A4, RRID:AB_2847875
Pluripotency Marker	Mouse anti-Sox-2, Cy3 conjugate	1:100	Millipore Sigma, MAB4423C3, RRID:AB_2847876
Pluripotency Marker	Mouse anti-Nanog, Alexa Fluor® 488 conjugate	1:100	Millipore Sigma, SCR078, MABD24A4, RRID:AB_2847877
Mesoderm marker	smooth muscle actin	1:200	Invitrogen, A25531, RRID:AB_2651005
Endoderm marker	α-fetoprotein	1:200	Invitrogen, A25530, RRID:AB_2651004
Ectoderm marker	β-III tubulin	1:200	Invitrogen, A25532, RRID:AB_2651003
Primers			
	Target	Forward/Reverse primer (5′-3′)	

Mycoplasma testing	Mycoplasma	Forward primers: CGCCTGAGTAGTACGTTCGC; CGCCTGAGTAGTACGTACGC; TGCCTGAGTAGTACATTCGC; TGCCTGGGTAGTACATTCGC; CGCCTGAGTAGTATGCTCGC/ Reverse primers: GCGGTGTGTACAAGACCCGA; GCGGTGTGTACAAAACCCGA; GCGGTGTGTACAAACCCCGA	
Mutation analysis for HT188	*PARK7*	TTCTGTGCTTTTGCCAGATG/TCTTTAGCAAGAGGGTGTGTTG	
CRISPR diagnostic primers	Donor oligo	TGGGCTCAAGCAATTTTTCTA/ATGGAGCGAGACTCCATCT	
gRNA for A18945	PARK7 mutation	caccgCTTAGGTCCTACTGCTCTGT/ aaacACAGAGCAGTAGGACCTAAGc	
Donor oligo		TGCGATTTTTTAAACATGGGCTTTTCTATATCTGCACTTAGAT	
		CTTTTTATTTTTATTCTTAGGTCCTACTCTCTGTTGGCTCATGA	
		AATAGGTTTTGGAAGTAAAGTTACAACACACCCTCTTGCTAA	
		AGACAAAATGATGAATGGAGGTAAGTATATGCTTGTTTTTGT	
		TTGTTTGTTTGTTTTTTGAGATGGAGTC	
