# Supplementary material for: Generation of iPSC line from a Parkinson patient with PARK7 mutation and CRISPR-edited Gibco human episomal iPSC line to mimic PARK7 mutation
Source: Stem Cell Res. Author manuscript; Available in PMC 2021 Sep 20. (PMC8451958; doi:10.1016/j.scr.2021.102506)
Supplement: 1 [file NIHMS1739539-supplement-1.pdf]

Supplemental Figure 1

A

Patient line | Control line | Edited line

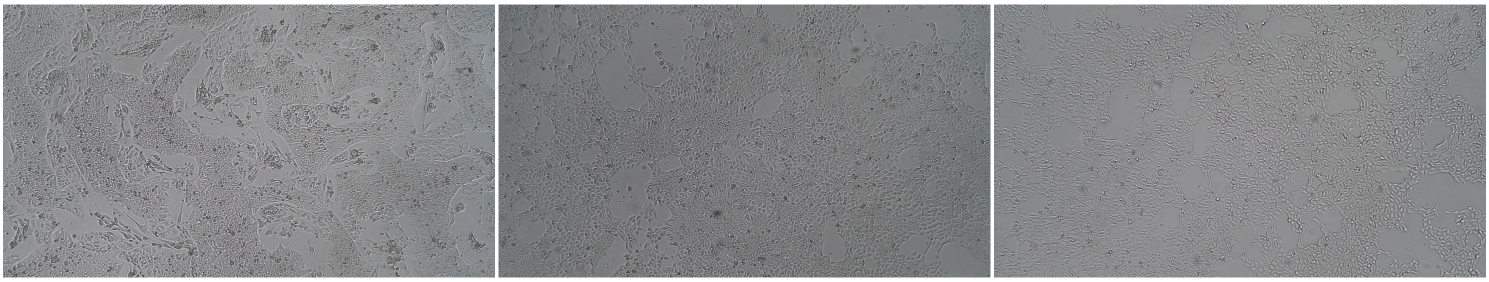

B

Patient line | Control line | Edited line

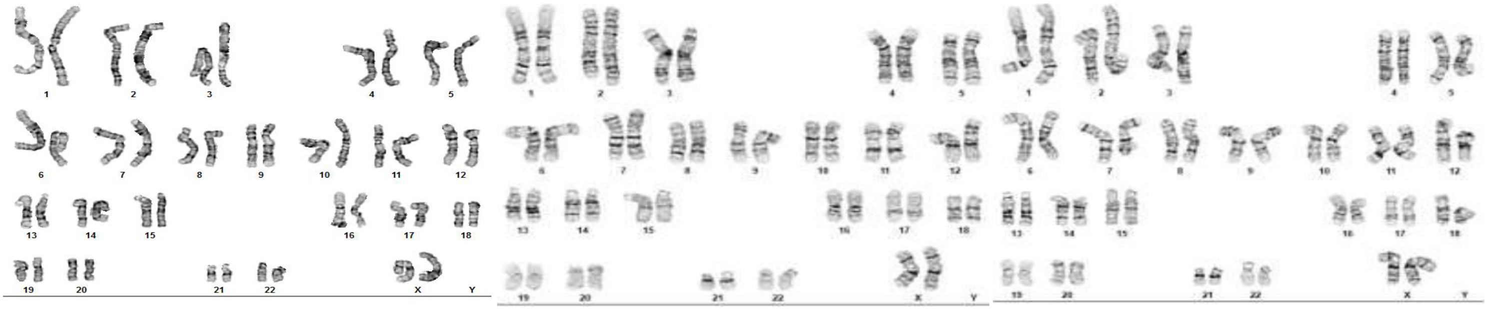

C

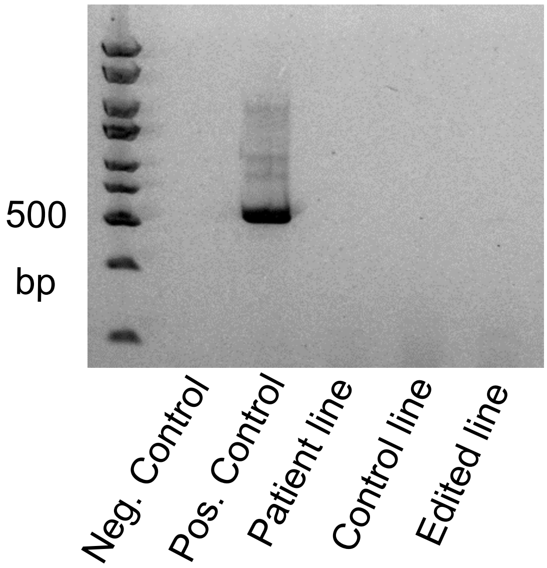

D

Chromosome	Position	Sequence	Strand	Mismatches
chr1	35931977	CTTAGGcCaTACTGCaCTGT(GGG)	+	3
chr1	244191886	CTTAGtTCCTACTGCTtTGc(TGG)	-	3
chr2	162266236	CTTtGccCCTACTGCTCTGT(TGG)	-	3
chr8	104220814	CTTAGGaCCTAaTGCTCTGc(AGG)	+	3
chr15	65034637	CTTtGGggCTACTGCTCTGT(GGG)	-	3
chr19	28389307	CTctGGTCCTgCTGCTCTGT(AGG)	+	3
chrY	8798586	CTcAGGTCCTACaGCTCTtT(GGG)	+	3
